# *Parvimonas micra* enhance gingpain activity in multi-species consortia

**DOI:** 10.1080/20002297.2017.1325189

**Published:** 2017-06-09

**Authors:** Jessica Neilands, Floris Bikker, Gunnel Svensäter

**Affiliations:** ^a^ Malmö University, Malmö, Sweden; ^b^ ACTA University of Amsterdam, Amsterdam, the Netherlands

## Abstract

Dental biofilms are complex and consists of many different species which can interact with each other and affect virulence properties. In this study we investigated the effect of *Parvimonas micra* on proteolytic activity in a multi-species bacterial consortium.

Multispecies consortia were constructed, with and without *P. micra*, using the following species: *Porphyromonas gingivalis, Prevotella intermedia, Fusobacterium nucleatum, Actinomyces naeslundii, P. micra, Streptococcus oralis, Streptococcus mitis, Streptococcus gordonii* and *Streptococcus cristatus*. The bacteria were grown anaerobically in 10% serum. Proteolytic activity was measured after 24h and 7 days using fluorogenic substrate, BIKKAM-10 specific for gingipains. The gingipain activity was expressed as relative fluorescent units/min/10^7^ cells. In addition, the effect of supernatants from over-night grown *P. micra* on gingipain activity was investigated in a single species *P. gingivalis* model.

After 24h the gingipain activity in the consortium was low and there was no significant difference between the two consortia. However after 7 days, the gingipain activity was 7-fold higher in the consortium with *P. micra* present. Twenty-four hour exposure of *P. gingivalis* to *P. micra* supernatants resulted in a 3-fold increase in gingipain activity compared to control cells.

The presence of *P. micra* enhance the activity of *P. gingivalis* gingpains. Further studies revealed that secernated products of *P. micra* were involved.

